# The impact of smoking on outcomes following anterior cervical fusion-nonfusion hybrid surgery: a retrospective single-center cohort study

**DOI:** 10.1186/s12891-021-04501-4

**Published:** 2021-07-09

**Authors:** Han Wang, Yang Meng, Hao Liu, Xiaofei Wang, Ying Hong

**Affiliations:** 1 Department of Orthopaedic Surgery, West China Hospital, Sichuan University, No. 37 Guo Xue Road, Sichuan 610041 Chengdu, China; 2 Department of Anesthesia and Operation Center, West China Hospital, Sichuan University, Sichuan, China; 3 West China School of Nursing, West China Hospital, Sichuan University, Sichuan, China

**Keywords:** Cervical disc replacement, Anterior cervical discectomy and fusion, Hybrid surgery, Heterotopic ossification, Bone loss, Smoking

## Abstract

**Background:**

There is mixed evidence for the impact of cigarette smoking on outcomes following anterior cervical surgery. It has been reported to have a negative impact on healing after multilevel anterior cervical discectomy and fusion, however, segmental mobility has been suggested to be superior in smokers who underwent one- or two-level cervical disc replacement. Hybrid surgery, including anterior cervical discectomy and fusion and cervical disc replacement, has emerged as an alternative procedure for multilevel cervical degenerative disc disease. This study aimed to examine the impact of smoking on intermediate-term outcomes following hybrid surgery.

**Methods:**

Radiographical and clinical outcomes of 153 patients who had undergone continuous two- or three-level hybrid surgery were followed-up to a minimum of 2-years post-operatively. The early fusion effect, 1-year fusion rate, the incidence of bone loss and heterotopic ossification, as well as the clinical outcomes were compared across three smoking status groups: (1) current smokers; (2) former smokers; (3) nonsmokers.

**Results:**

Clinical outcomes were comparable among the three groups. However, the current smoking group had a poorer early fusion effect and 1-year fusion rate (*P* < 0.001 and *P* < 0.035 respectively). Both gender and smoking status were considered as key factors for 1-year fusion rate. Upon multivariable analysis, male gender (OR = 6.664, 95% CI: 1.248–35.581, *P* = 0.026) and current smoking status (OR = 0.009, 95% CI: 0.020–0.411, *P* = 0.002) were significantly associated with 1-year fusion rate. A subgroup analysis demonstrated statistically significant differences in both early fusion process (*P* < 0.001) and the 1-year fusion rate (*P* = 0.006) across the three smoking status groups in female patients. Finally, non-smoking status appeared to be protective against bone loss (OR = 0.427, 95% CI: 0.192–0.947, *P* = 0.036), with these patients likely to have at least one grade lower bone loss than current smokers.

**Conclusions:**

Smoking is associated with poor outcomes following hybrid surgery for multilevel cervical disc disease. Current smokers had the poorest fusion rate and most bone loss, but no statistically significant differences were seen in clinical outcomes across the three groups.

## Background

Anterior cervical discectomy and fusion (ACDF) is a traditional surgical procedure for the treatment of cervical degenerative disc disease (CDDD). Although satisfactory postoperative clinical outcomes have been reported from this procedure, non-fusion remains a concern. Previous literature suggests that the fusion rate may decrease with number of segments involved, the fusion rate for three-level ACDF has been reported as low as 56% up to 37 months after surgery [1]. In addition, multilevel fusion surgery decreases the cervical range of motion (ROM), leading to more pressure across adjacent levels. This may increase the risk of adjacent segmental degeneration (ASD). Cervical disc replacement (CDR) has therefore gained widespread popularity to preserve segmental mobility and mitigate against the risk of ASD. However, to date, Mobi-C and Prestige-LP artificial cervical discs are approved for use only in double-level CDDD by the Food and Drug Administration, although three-level CDR has been successfully performed, it is still considered an experimental treatment [2, 3]. Hybrid surgery (HS), which combines ACDF and CDR has been proposed to mitigate these concerns. The objective of HS is to tailor the optimal surgical procedures to each target level according to its degenerative status. As a result, several series have demonstrated that this is a safe and effective surgical procedure for the treatment of multilevel CDDD [4–7].

Cigarette smoking is known to be associated with several health problems, including asthma, diabetes, cardiovascular disease, and other debilitating conditions. Nearly one billion people will die from smoking-related issues during the twenty-first century [8]. Smoking has also been demonstrated to worsen bone health, with an increased risk of osteoporotic fractures and delayed fracture healing in current smokers. A meta-analysis of 40,753 patients showed that smoking increases the risk of hip fracture by 30-40% [9]. A second meta-analysis of 59,232 patients reported a 25% increase in overall fractures and 84% increase in hip fractures in smokers compared with nonsmokers [10]. Moreover, several studies have reported a myriad of deleterious effects of smoking on patients undergoing spine surgery including increased complications, lower fusion rates, poorer clinical outcomes, and decreased quality of life [11–16]. Hilibrand et al. monitored 190 patients over two years and found smoking to be associated with a significant negative effect on healing and clinical recovery after multilevel cervical ACDF with autogenous interbody graft [17]. Contradictory to this, Tu et al. found that segmental mobility was marginally improved in smokers in patients with one- and two-level CDR than non-smokers [18]. However, the effect of smoking on anterior cervical HS is unclear. The adverse effects of smoking on arthrodesis and the potential benefits in arthroplasty contradict one another in the context of a hybrid approach.

In the present study, we aimed to explore the clinical impact of smoking status on fusion and bone loss in patients undergoing two- and three-level HS. To date, this is the first study to address this issue. We also hope to explore the impact of smoking on postoperative outcomes of HS to improve the evidence for implementation.

## Method

### Patient population

This retrospective study was conducted in according with the approval of our institutional review board. Consecutive patients who had undergone two- and three-level HS using the Prestige-LP artificial cervical disc (Medtronic, Minneapolis, Minnesota) and Zero-P device (Synthes GmbH Switzerland, Oberdorf, Switzerland) in our institution were included in this analysis. The inclusion criteria were as follow: (1) radiological findings consistent with foramen stenosis, ossification of posterior longitudinal ligament, obvious osteophytes on X-ray or CT scan or herniated nucleus pulposus on MRI; (2) symptomatic cervical myelopathy or radiculopathy or both; (3) failed conservative management for 6 weeks or more; (4) patient provided informed consent to undergo consecutive HS. The exclusion criteria were as follows: (1) less than 24 months of complete follow-up; (2) incomplete clinical and radiological data; (3) other indicators for cervical surgery such as spinal trauma, tumors, infection; (4) previous cervical spine surgery.

### Surgical technique

The selection of either arthroplasty or arthrodesis during HS has strict indications. CDR was performed at levels without sagittal plane translation > 3 mm or sagittal plane angulation > 11°; without the ROM < 3°; without a disc height loss > 50%; and without facet joint degeneration, bridge osteophytes, or instability. ACDF was performed at the levels that did not meet the above criteria.

All operations were performed by the same senior spine surgeon. The patient was placed on the back with the neck in the neutral position. A standard right-side approach to the anterior cervical spine was adopted. After sufficient decompression of the entirety of the intervertebral space, CDR was performed before ACDF where indicated. A prothesis of the most suitable size was implanted into the intervertebral space, and the same artificial bone tissue was used in all the arthrodesis levels. Finally, C-arm fluoroscopy was used to confirm the appropriate position of the implants.

### Clinical and radiological evaluations

**Fig. 1:**
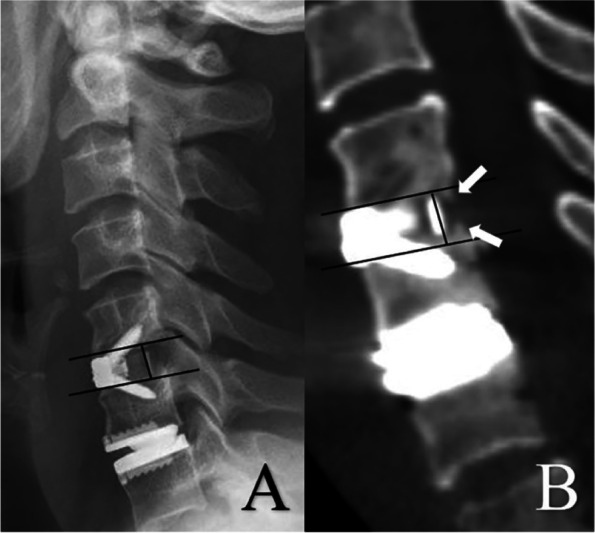
A lateral X-ray at 3-month follow-up was used to confirm the shape of endplates and the location of the marker line (**A**). The height of bone tissue along the posterior border of the cage was measured in the sagittal CT scan as the reflection of early fusion effect. (The white arrows in **B**)

Baseline demographics, clinical and radiological data were collected for all patients. Clinical outcomes were evaluated by the Japanese Orthopedic Association (JOA) scale, Neck disability Index (NDI), and visual analog scale (VAS) for neck and arm pain both preoperatively and at the final follow-up appointment. Pre- and postoperative radiological evaluations included X-ray, CT, and MRI. Lateral and extension-flexion radiographs, sagittal reconstructed CT, and T2-weighted MRI were obtained at specified time points. Segmental ROM at the target level and the ROM of C2-7 was defined as the difference in the Cobb angle between extension and flexion radiographs. For patients with two-level CDR, the mean value of segmental ROM was used for further analysis. Bone mineral density (BMD) was measured at the L2-4 vertebral body. Heterotopic ossification (HO) was using the McAfee classification [19] (Table 1). The classification of bone loss (BL) followed a validated methodology detailed in full elsewhere, based on a classification system reported by Saleh et al. in 2004 [20, 21] (Table 2). For patients with two-level CDR, the more severe degree of HO and BL was recorded. McAfee grade III and IV HO were classified as high-grade HO. Early fusion process was assessed using sagittal reconstructed CT scans at three-month follow-up, measuring the height of new bone tissue at the posterior aspect of the cage (Fig. 1.) For patients with two-level ACDF, the mean value of new bone tissue was used for further analysis. The criteria to confirm fusion at 1-year were segmental ROM less than 3° in X-ray and continuous bone bridge demonstrated in CT imaging. For patients with two-level ACDF, if both levels had achieved fusion they were classified as “fusion”, otherwise they were classified as “non-fusion”. All measurements and ratings were completed by two independent spine surgeons, with corroboration by a third senior spine surgeon in case of disagreement.

**Table 1:** Classification of HO following CDR

Grade	Definition
0	No HO was observed
1	HO does not occur within the disc space
2	HO is present between the planes formed by the vertebral endplates but does not block spinal motion
3	The range of motion of the vertebral endplates is blocked by the formation of HO or osteophytes
4	HO causes bony fusion

*HO* heterotopic ossification, *CDR* cervical disc replacement

**Table 2:** Classification for BL after CDR

Grade	Definition
0	None. BL accounts for 0–1% of the length of endplate
1	Mild. BL accounts for 1–5% of the length of endplate
2	Moderate. BL accounts for 5–10% of the length of endplate
3	Severe without collapse. BL accounts for > 10% of the length of endplate without prosthesis subsidence
4	Severe with collapse. BL accounts for > 10% of the length of endplate with prosthesis subsidence

*BL* bone loss, *CDR* cervical disc replacement

### Study group and statistical analysis

The main explanatory variable for this study was smoking status. All patients were classified into one of three groups according to their smoking status at the time of surgery: (1) patients that had smoked cigarettes within the year prior to surgery were defined as the current smokers; (2) patients with a smoking history but with cessation of smoking for more than 1 year before surgery were defined as former smokers; (3) patients who had never smoked were defined as non-smokers. All patients were recommended to stop smoking postoperatively. Continuous variables were summarized using the mean average and standard deviation. Frequency data is presented as quantities and proportion. Statistical analysis was performed using SPSS 19.0 for windows (IBM SPSS Inc, New York, USA). ANOVA analysis was used to compare continuous data across the three explanatory groups and Fisher’s least significant difference (LSD) test was used for pairwise comparisons. Inter- and intra-observer reliability were assessed using intra-class correlation coefficient (ICC). Kruskal–Wallis H test was used to explore factors associated with bony fusion, and multiple ordered regression was used to identify the risk factors associated with BL. Both were presented as adjusted odds ratio (OR) with 95% confidence intervals (95% CI). The alpha level of significance was set at 0.05.

## Results

### Baseline demographics

A total of 153 patients with complete clinical and radiological data were included in analyses. Of these, 51 (33.33%) were male and 102 (66.67%) were female. The mean age was 50.11 years old, with a mean follow-up of 40.82 months. In total, 79 patients underwent 2-level HS and 74 underwent 3-level HS. Other demographics data are summarized in Table 3.

**Table 3:** Baseline demographic and clinical characteristic

Variable	Value (%)
Number of patients	153
Age (years)	50.11 ± 7.48
Sex	
Male	51
Female	102
Involved levels	
Two	79
Three	74
T value (spine)	0.30 ± 1.03
Follow-up (months)	40.82 ± 15.63
HO formation	92(58.97%)
High-grade HO formation	16(10.26%)
Bone loss	84(53.85%)

*HO* heterotopic ossification

### Inter- and intra-observer differences

The inter- and intra-observer reliability were 0.94 and 0.91 for preoperative segmental ROM, 0.87 and 0.92 for preoperative C2-7 ROM, 0.92 and 0.96 for postoperative segmental ROM, 0.92 and 0.89 for postoperative C2-7 ROM, 0.98 and 0.97 for HO, 0.91 and 0.96 for bone loss, 0.95 and 0.91 for early fusion effect, 0.97 and 0.95 for 1-year fusion rate.

### Smoking status

**Fig. 2:**
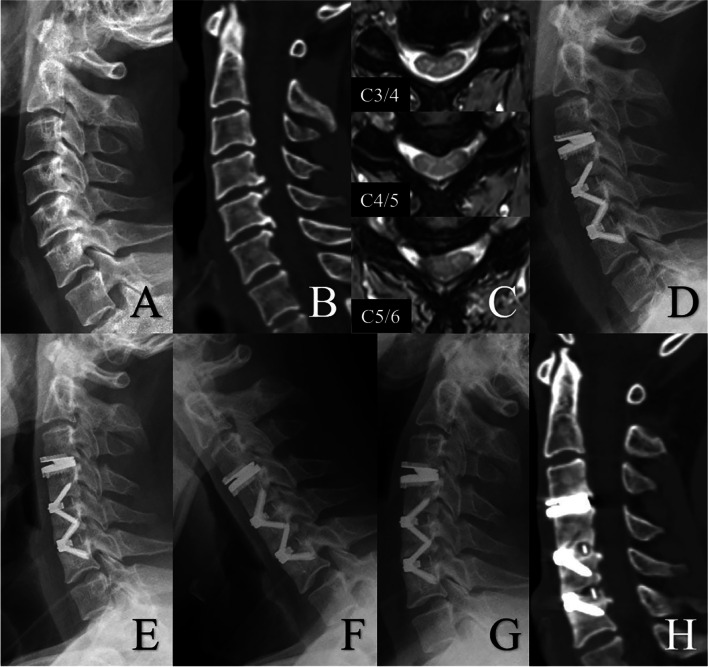
Radiologic examinations of a 45-year older woman with neck pain for 1 year, who had cigarette consumption for more than 10 years. Preoperative lateral X-ray showed good cervical lordosis (**A**). However, a sagittal CT scan showed osteophytes at the posterior border of C4/5 and C5/6 (**B**). MRI demonstrated spinal cord compression at C3/4, C4/5 and C5/6 (**C**). The patient underwent HS, including CDR at C3/4, and ACDF at C4/5 and C5/6 **(D**). At 1-year follow-up, lateral X-ray shows satisfactory cervical lordosis (**E**), and extension-flexion view showed good cervical ROM (**F** and **G**). However, a postoperative CT scan showed incomplete bony fusion at both two arthrodesis levels (**H**)

There were 40 (26.14%) current smokers, 34 (22.22%) former smokers and 79 (51.63%) non-smokers included in this study. Men were most likely to be current smokers while women were most likely to be nonsmokers (47.06% vs 66.67%, *P* < 0.001). The BMD value in the nonsmoking group was numerically higher than those of other two groups, but this was not statistically significant. The clinical and radiological outcomes by the point of final follow-up were comparable across all three groups. No statistical differences were found in the incidence of HO and BL, but the current smoking group had the worst early fusion effect (*P* < 0.001) and lowest 1-year fusion rate (*P* < 0.035) (Table 4) Upon multivariable analysis, both gender and smoking status were associated with 1-year fusion rate (Fig. 2). Male patients (OR = 6.664, 95% CI: 1.248–35.581, *P* = 0.026) displayed increased odds whilst non-smokers demonstrated reduced odds (OR = 0.009, 95% CI: 0.020–0.411, *P* = 0.002) (Table 5). Subgroup analysis was therefore used to further explore the effect of gender and smoking status on the postoperative outcomes. Although there were significant differences in early fusion process among the three smoking status groups for male group (*P* < 0.001), no significant differences were observed in 1-year fusion rate (Table 6). However, for female patients, statistical differences were found in both early fusion process (*P* < 0.001) and the 1-year fusion rate (*P* = 0.006) (Table 7). Additionally, no significant differences were observed in the degree of HO among the three smoking status groups, however, the degree of BL in current smokers was found to be the most severe (*P* < 0.001) (Table 8). Reinforcing this, multiple ordered regression demonstrated that sex was not associated with BL, whilst smoking status was significantly associated (Table 9). Current smokers demonstrated the most serious BL, and patients who had never smoked were likely to have at least one grade lower BL than current smokers (OR = 0.427, 95% CI: 0.192–0.947, *P* = 0.036).

**Table 4:** Comparison of clinical and radiological data among current smokers, former smokers and non-smokers

	Current smoker	Former smoker	Nonsmoker	*P* value
Number of patients	40	34	79	
Sex (M:F)				< 0.001*
Male	24	16	11	
Female	16	18	68	
Age (years)	50.00 ± 6.08	51.62 ± 7.40	49.52 ± 8.13	0.393
Follow-up (months)	52.10 ± 13.95	47.71 ± 14.82	47.65 ± 16.69	0.306
T value (spine)	0.24 ± 1.01	0.26 ± 1.19	0.34 ± 0.97	0.861
Preoperative outcomes				
JOA	10.60 ± 1.03	11.12 ± 1.59	10.80 ± 1.39	0.261
NDI	30.50 ± 3.63	30.47 ± 3.86	31.37 ± 3.87	0.362
VAS-arm	5.93 ± 1.00	5.44 ± 1.37	5.68 ± 1.20	0.224
VAS-neck	5.98 ± 0.83	5.68 ± 1.12	5.90 ± 1.08	0.435
Segmental ROM	9.48 ± 5.11	11.25 ± 4.76	10.13 ± 4.51	0.273
C2-7 ROM	49.16 ± 13.81	53.44 ± 12.66	48.99 ± 12.16	0.210
Involved levels				0.116
C3/4	14	10	7	
C4/5	38	28	59	
C5/6	40	30	76	
C6/7	22	14	42	
Final outcomes				
JOA	15.55 ± 0.81	15.79 ± 1.23	15.76 ± 1.05	0.511
NDI	8.48 ± 2.61	7.85 ± 3.09	7.86 ± 2.59	0.467
VAS-arm	1.63 ± 0.67	1.44 ± 1.05	1.63 ± 0.89	0.544
VAS-neck	1.50 ± 0.51	1.59 ± 0.50	1.48 ± 0.60	0.636
Segmental ROM	7.33 ± 3.53	7.58 ± 4.09	8.17 ± 4.04	0.508
C2-7 ROM	36.26 ± 10.65	36.70 ± 11.38	37.47 ± 10.89	0.838
Heterotopic ossification	24	22	46	0.812
Bone loss	22	22	40	0.386
Early fusion effect	2.35 ± 0.73	3.20 ± 1.59	3.62 ± 1.41	< 0.001*
1-year fusion rate	33	32	76	0.035*

^*^P < 0.05, statistically significant

**Table 5:** Factors associated with one-year fusion status in the binary logistic regression model

	P value	OR value
Male	0.026*	6.664(1.248–35.581)
Female	-	
Current smoker	0.002*	0.090(0.020–0.411)
Former smoker	0.360	
Nonsmoker	-	

^*^P < 0.05, statistically significant

**Table 6:** The impact of smoking on male anterior cervical HS patients

	Current smoker	Former smoker	Nonsmoker	*P* value
Number of patients	24	16	11	
Final Outcomes				
JOA	15.50 ± 0.83	15.94 ± 1.39	16.00 ± 1.18	0.335
NDI	8.04 ± 1.57	7.81 ± 3.47	6.55 ± 0.93	0.192
VAS-arm	1.58 ± 0.65	1.31 ± 1.25	1.55 ± 0.52	0.611
VAS-neck	1.50 ± 0.51	1.63 ± 0.50	1.55 ± 0.52	0.750
Segmental ROM	6.42 ± 2.81	6.98 ± 4.20	7.83 ± 3.14	0.520
C2-7 ROM	36.29 ± 9.92	35.09 ± 9.36	34.56 ± 10.46	0.870
Early fusion effect	2.63 ± 0.57	4.10 ± 1.66	5.35 ± 2.18	< 0.001*
1-year fusion rate	22	16	11	0.699

*HS* hybrid surgery, *JOA* Japanese Orthopaedic Association, *NDI* neck disability index, *VAS* visual analog scale, *ROM* range of motion. *P < 0.05, statistically significant

**Table 7:** The impact of smoking on female anterior cervical HS patients

	Current smoker	Former smoker	Nonsmoker	*P* value
Number of patients	16	18	68	
Final Outcomes				
JOA	15.63 ± 0.81	15.67 ± 1.08	15.72 ± 1.03	0.935
NDI	9.13 ± 3.63	7.89 ± 2.81	8.07 ± 2.71	0.374
VAS-arm	1.69 ± 0.70	1.56 ± 0.86	1.65 ± 0.94	0.901
VAS-neck	1.50 ± 0.52	1.56 ± 0.51	1.47 ± 0.61	0.857
Segmental ROM	8.70 ± 4.12	8.12 ± 4.04	8.22 ± 4.18	0.902
C2-7 ROM	36.23 ± 12.00	38.14 ± 13.02	37.95 ± 10.96	0.853
Early fusion effect	1.92 ± 0.75	2.34 ± 1.02	3.34 ± 1.02	< 0.001*
1-year fusion rate	11	16	65	0.006*

*HS* hybrid surgery, *JOA* Japanese Orthopaedic Association, *NDI* neck disability index, *VAS* visual analog scale, *ROM* range of motion. *P < 0.05, statistically significant

**Table 8:** Comparison of degree of HO and BL among the current smokers, former smokers and non-smokers

	Current smoker	Former smoker	Nonsmoker	*P* value
Heterotopic ossification	24	22	46	0.097
1	0	4	6	
2	18	14	34	
3	0	4	2	
4	6	0	4	
Bone loss	22	22	40	< 0.001*
1	10	12	39	
2	6	9	0	
3	6	1	1	
4	0	0	0	

*HO* heterotopic ossification, *BL* bone loss. *P < 0.05, statistically significant

**Table 9:** Factors associated with bone loss in the multiple ordered logistic regression model

	*P* value	OR value
Nonsmoker	0.036*	0.427(0.192–0.947)
Former smoker	0.737	
Current smoker	-	
Female	0.593	
Male	-	

^*^P < 0.05, statistically significant

## Discussion

HS, including both CDR and ACDF, is now one of the most common surgical procedures for the treatment of patients with cervical spondylosis. Previous studies have suggested that smoking may limit bony fusion following ACDF, but has a potential advantage in the ROM permitted after CDR. For the patients undergoing a hybrid approach, evidence is conflicting, with an unclear effect of smoking where both arthroplasty and arthrodesis are performed. To the best of our knowledge, this is the first study that focuses on this issue, it aimed to explore the impact of smoking on clinical outcomes and complications after HS.

The negative impact of smoking on bone health has been well described, including increased rates of osteoporosis, osteoporotic fracture and bone loss. The Nurses’ Health Study, a prospective cohort of 121,701 female nurses aged between 30–55 years old, found that current smokers had a dose-independent increase in the incidence of hip fracture compared with non-smokers [22]. A Norwegian cohort study of 34,856 adults aged more than 50 years old showed smoking was associated with the incidence of the hip fracture in both sexes, and that effect was independent off body mass index and physical inactivity [23]. Additionally, a community-based, longitudinal, epidemiologic study of osteoporosis in 1789 people over the age of 60 showed smoking was associated with 5–8% lower BMD at the spine and the femoral neck [24]. The present study corroborated these findings, demonstrating that BMD was higher in the non-smokers than in current and former smokers. Our data suggest a degree of recovery of bone health in former smokers, with a mean BMD value between that of current and non-smokers. However, previous studies have reported that few differences in BMD was observed between current and former smokers [24, 25]. This warrants further investigation in future work.

In cervical spine arthrodesis, several studies have shown that smoking has a negative impact on healing following spinal fusion surgery [11, 15, 17]. This is supported by a considerable body of translational research devoted to exploring the mechanistic effects of smoking on bone healing. Chang et al. reported that cigarette smoking impairs angiogenesis in early bone healing process and delays fracture union [26]. Ueng et al. hypothesized that smoking delays mineralization during the bone healing process and further decreases the mechanical strength of the regenerating bone [27]. EI-Zawawy et al. found that smoking delays chondrogenesis during bone healing in a mouse model [28]. Supporting this, in our study less new bone tissue was measured at the posterior margin of the cage in the current smokers, compared with the former and non-smokers. In further laboratory work, Davies et al. reported nicotine to have deleterious effects on wound healing through increased vasoconstriction [29]. Gaston et al. put forward several hypotheses about the impact of smoking on bone healing process, including reduced blood supply, deficiency of vitamins and antioxidants, and high levels of reactive oxygen intermediates [30].

Gender is a potential confounding factor in this study, with a higher rate of current smokers than non-smokers in male patients, and a significantly higher fusion rate in male compared with female patients. To explore the effect of smoking on the bony fusion, subgroup analyses were performed by gender. No significant differences were observed in male patients across smoking status groups. For female patients in the current smoking group, a lower fusion rate was observed than that of former smokers and nonsmokers. One reason for this may be related to estrogen levels. The average age of included patients was around 50 years old, which commonly represents the perimenopausal period. A decrease in the circulating estrogen level may lead to a decrease in BMD and bony fusion. Additionally, it has been previously reported that smoking may lead to decreased bio-availability of estrogen in target tissues [31]. This may explain the reason why women in the current smoking group showed a poorer 1-year fusion rate. Although the reasons are likely to be multifactorial, our study broadly confirms that smoking has a negative impact on bone healing, and may impair early osteogenesis and fusion of arthrodesis levels in patients undergoing HS.

In terms of the effect of smoking on the arthroplasty levels, however, current research is very limited. To date, only a single retrospective study of 197 patients who underwent one- or two-level CDR has been reported. At an average of 3.5 years follow-up, Tu et al. reported similar clinical outcomes between current smokers and non-smokers, but with a slightly better segmental ROM observed in smokers [18]. In the present study, no significant differences were found in clinical outcomes or ROM across the three smoking status groups. However, the patients in the current smoking group had the highest level of BL among the three groups. Wang et al. performed a systematic review of six studies including 440 patients who underwent CDR across 536 segments. They found that patients with BL achieved similar clinical outcomes compared with those without BL [32]. Wu et al. performed a retrospective study of 396 patients in a single center and found BL did not affect clinical outcomes but patients with BL had a larger segmental ROM [33]. However, Hacker et al. reported that one patient with BL had recurrent neck and arm pain for 52 months postoperatively, with radiological evaluations demonstrating segmental kyphosis. This patient eventually required revision surgery and two-level ACDF [34]. Again, a wealth of basic research has demonstrated the impact of smoking on resorption and BL in animal models [35, 36]. Although the clinical outcomes associated with BL remain unclear, it should be recognized that severe BL can lead to prosthesis collapse, and a need for revision. Additionally, no significant differences were observed in the incidence of HO among the three smoking groups, but with a trend towards more severe HO in current smokers. Although HO is essentially an osteogenic process, its true mechanism is still unclear. Male gender, old age and multilevel diseases have been previously considered to be risk factors for HO [37, 38]. A meta-analysis of 94 studies indicated that the incidence of HO increased over time in studies with longer follow-up [39]. Another meta-analysis compared the incidence of high-grade HO across studies and found a significant variability between different prostheses [40]. Additionally, endplate coverage, segmental angle and center of rotation were all considered to be associated with the presence of HO [41–43]. However, the effect of smoking on HO requires further exploration.

In summary, smoking had a negative impact on patients undergoing HS. The patients in the current smoking group had a worse early fusion process and 1-year fusion rate, particularly in female patients, as well as more serious BL. Smoking cessation is recommended for all patients before HS. Additionally, all patients undergoing HS are required to wear a collar for at least three months in our institution, with supervised activity permitted during the first three weeks postoperatively. Considering the poor early fusion effect and stability in current smokers, protracted periods in a neck brace and reduced activity may be necessary for these patients in the early postoperative period. The present study has some limitations. First, the retrospective design may have led to potential selection bias. The dose and duration of smoking were also unavailable. Second, passive smoking history was not included as a factor in the multivariable models, which may have introduced unmeasured confounding. Third, the patient sample was small and the follow-up duration was short, with data only from a single institution. A multicenter study with a larger sample size and longer follow-up period would be important to provide stronger evidence.

## Conclusion

This study showed that smoking has a negative impact on the patients undergoing HS. Smoking cessation is important to consider for these patients before surgery in order to reduce risk and approach baseline. The patients in the current smoking group had a worse early fusion effect and 1-year fusion rate, as well as more serious BL. However, no significant differences were observed in clinical outcomes among the three groups.

## Data Availability

All data generated or analyzed during this study are included in this article.

## Abbreviations

ACDF: Anterior cervical discectomy and fusion
CDDD: Cervical degenerative disc disease
ROM: Range of motion
ASD: Adjacent segmental degeneration
CDR: Cervical disc replacement
HS: Hybrid surgery
JOA: Japanese Orthopaedic Association
NDI: Neck disability index
VAS: Visual analog scale
HO: Heterotopic ossification
BMD: Bone mineral density
BL: Bone loss
ICC: Intra-class correlation coefficient
